# End-User Skin Analysis (Moles) through Image Acquisition and Processing System

**DOI:** 10.3390/s22031123

**Published:** 2022-02-01

**Authors:** Lorant Andras Szolga, Denisa Alice Bozga, Camelia Florea

**Affiliations:** 1 Basics of Electronics Department, Technical University of Cluj-Napoca, 400114 Cluj-Napoca, Romania; bozga.ma.denisa@student.utcluj.ro; 2 Communications Department, Technical University of Cluj-Napoca, 400114 Cluj-Napoca, Romania; Camelia.Florea@com.utcluj.ro

**Keywords:** ABCD rule, microcontroller, image processing, mole, photography, skin cancer

## Abstract

Skin moles and lesions can be the first signs of severe skin diseases such as cancer. This paper presents the development of an end-user device capable of capturing images, segmentation and diagnosis of moles by using the ABCD rule, which stands for analyzing moles’ parameters as: asymmetry, border, color, and diameter. These are the main mole characteristics that doctors look at, each of them having a different factor of importance, and depending on these an accurate diagnosis can be given. For the hardware, we developed a small and compact device that can be manipulated easily by anyone without knowledge of medicine, in which we considered a custom-designed 3D enclosure with two white LEDs to control the light. The device has the role of facilitating analysis of the suspicious moles regularly at home, even if only from an indicative and not from a medical point of view. The developed PC software permits the storage of the images in a local database for easy tracking and analysis in time. The image processing developed for the ABCD rule is incorporated into the PC software and tested extensively on the international PH2 database with skin melanoma images to validate our segmentation and criteria evaluation. Using the developed device, we captured mole images for patients, who also took a medical examination by a specialist using the standard dermatoscope. Therefore, we obtained our own database containing 26 images for which we have also the specialists’ diagnosis. The performance evaluation measures obtained using our device are—Accuracy: 0.92, Precision: 1.0, Recall: 0.92, F1-score: 0.96.

## 1. Introduction

The skin is the largest organ of the body [1] and represents much more than a simple covering. It is a complex structure of tissues that work together to protect the body in many ways. The nerve endings on its surface transmit information about the environment to the brain. The most well-known skin disease is, by far, skin cancer. It is one of the most common forms of cancer, is growing globally, and is expanding due to the development of abnormal growth in cells, irreparable damage to skin cell DNA caused mainly (>90%) by UV radiation.

Historically, the diagnosis of skin cancer has depended on different conventional techniques. A variety of commercial diagnostic tools and auxiliary techniques have been available since 1980 [2,3].

Today there are many methods for detecting and diagnosing skin cancer such as ultrasound systems [4], microscopy, Raman spectroscopy [5], fluorescent spectroscopy, Terahertz spectroscopy, and thermography [2].

Total Body Photography (TBP) is also a method for the early diagnosis of skin cancer. This method depends on the techniques of photography born in the middle of the 19th century and has proved to be a valuable tool in a wide range of fields: medicine, science, etc. The camera used to capture the first images was analogic. Its film had to be processed, which complicated the acquisition of images. The whole process came to be a long one, but part of important innovation in dermatology [6].

Sony was the first to launch in August 1981 the 0.3 Megapixel portable electronic camera. This camera was built as a ‘‘point-and-’shoot’ type. It used a CCD (Charge-coupled-device) sensor, which consists of a panel of photosensitive diodes that capture the image data together with a storage panel that picks up the images and represents an analog-moving register. This allows the analogic signals (electric charge) to be transported in several successive steps under the control of a clock signal [7].

The original analog camera was replaced by a digital camera thanks to developments in technology. This could make 2D images with increased resolution. The time for obtaining the images was much lower due to the improved processors and the cameras’ sensors that acquired an optical image and made this digital, which was made possible by converting the light into an electronic signal.

These sensors can be made via two technologies [8]:

- Charge-coupled-device (CCD).
- Complementary metal-oxide-semiconductor (CMOS).

Since 2015, a TBP 3D prototype (Vectra WB360, Canfield Scientific INC, Parsippany, NJ, USA) has been created and consists of 46 rooms. This prototype would eliminate the inconvenience of the multiple images made with 2D analysis. In 2017 a new 3D TBP system was launched, consisting of 92 cameras, which could create a 3D profile of the subject in seconds. 3D medical photography is still of high interest, and promises to be a fundamental tool for different fields of medicine. In the future, we will have increasingly versatile, smaller, and high-precision equipment [9].

As far as dermatology is concerned, the next step in developing the early diagnosis of skin cancer, and implicitly in the TBP technique, is the implementation of a more eloquent smartphone application with a user-friendly interface for high-risk moles.

Total body photography has been proven to be a valuable tool that can improve the early detection of skin cancer, as it observes changes in various lesions by the compression of images taken by an analog or digital camera, a 3D system, or an increasingly present smartphone application.

The ultimate objective of all these procedures is “No one should die of malignant melanoma“—AB Ackerman, 1990 [10].

Multiple studies show that people are quite reserved regarding medical check-ups for moles. Skin moles and lesions are the first signs that can lead to severe skin diseases such as cancer. This disease is on the rise globally.

This paper presents the development of an end-user device capable of diagnosing and classifying moles by the ABCD rule introduced in 1985 [11,12,13], which stands for evaluating the parameters: asymmetry, border, color, and diameter. Each criterion has a different factor of importance and, depending on these, an accurate diagnosis can be given. These are the main skin damage characteristics that doctors look for in the first step.

Image processing is a domain that is evolving rapidly. Its applications are met everywhere: medicine, the army, industry, art, or where information from the environment is represented in the form of images. For mole evaluation, image processing brings a strong advantage, helping doctors to numerically quantify the mole shape and the presence of colors in it—values that are very important in making a decision regarding a possibly cancerous case. In the state of the art, there are many papers dealing with this problem, some using the classical procedure of image shape analysis [12,14,15], and some implementing ML (Machine Learning) and DL (Deep Learning) methods [16,17,18]. Still, the ABCD rules are best as preferred and optimal methods for feature extraction, as can be seen from such recent works as [12,14,15,16,18], some of these combining the ABCD rule with other feature extraction techniques and applying ML for classification [16,18].

The proposed device has all the necessary elements to make a very accurate analysis: from image acquisition to image processing and user-friendly visualization of the result. Critical parameters for the hardware were considered, such as good quality images that are not affected by the ambient light conditions, and a small and compact device that can be manipulated easily by anyone without knowledge in medicine. Image capturing by different phone cameras in different light conditions alter the image processing quality, which implies customization of such software for each type of camera. The D parameter in such phone cameras must be measured independently by the user and introduced manually into the algorithm. With our dedicated hardware, the above drawbacks are eliminated. To save the captured images, a PC is needed with a USB connection. The developed PC software permits the storage of the images in a local database for easy tracking and analysis in time. The image processing developed for the ABCD rule is incorporated into the PC software and has been tested extensively on the PH2 international database with skin melanoma images to offer a high calibration standard for the images captured with the standalone device.

The proposed developed device comes to the aid of those who are afraid to, or do not have the time to, consult a specialist and has the role of facilitating the analysis of the suspicious moles regularly at home, even if only from an indicative and not from a medical point of view.

Using the developed device, we captured 26 images with moles for patients who also took a medical examination via a dermatoscope. The performance evaluation measures obtained using our device are—Accuracy: 0.92, Precision: 1.0, Recall: 0.92, F1-score: 0.96.

## 2. System Implementation

### 2.1. System Architecture

Our design system consists of a hardware and a software assembly. The electronic part of the hardware is developed around the OV7670 CMOS camera and the ATmega328 microcontroller. To ensure the proper functioning of image capturing without ambient light disturbance, and at the same time a friendly manipulation of the device, the electronic assembly was mounted in a custom-designed 3D enclosure with two white LEDs embedded. A USB connection to a PC is used to save the captured images. To analyze the captured images by the ABCD rule and to give a diagnosis based on this rule, Windows OS software was developed. The software interface allows the user to track the history of the captured moles. Thus, the end-user must connect the device to the USB port of the PC, place the opening of the 3D enclosure on the mole of interest, capture the image of the mole by a simple push of a button, and open the file in the PC software.

### 2.2. Hardware Design

**Figure 1:**
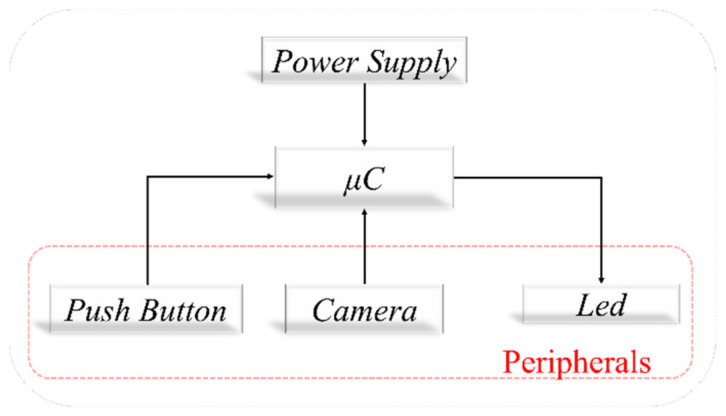
Block diagram of the hardware device.

The components we used in the hardware implementation of our device are an OV7670 camera module, Arduino Nano development board, two white LEDs, and one push-button. Figure 1 shows the block diagram of the entire device:

We chose the OV7670 or VGA camera module because it is small, has a low operating voltage, is based on the CMOS image sensor OV7670, and offers all the features of the single-chip VGA camera.

The VGA image is up to 30 frames/sec, allowing complete image quality control, data format, and mode of transmission. The image processing functions, including gamma curves, white balance, saturation, and chroma, can be programmed via the SCCB interface, which is compatible with the I2C serial communication protocol.

Obtaining images can be achieved in different ways. Initially, we used the Arduino Uno development board to make the prototype and then extracted the microcontroller to use on the printed circuit board, because one of the purposes is to make a device of small dimensions.

Since the transmission of images to the PC is done via USB, we used the Arduino Nano development board which is small in size and contains both the Atmega328P microcontroller and the Mini-B type USB.

It can be powered in three ways:

- By connecting the USB to either a charger or a PC.
- Through the Vin pin that can be powered from a source with a value between 6V and 12V, and with the help of the implemented regulator with an operating voltage of 5V [19].
- By connecting a 5V source to the pin for this voltage.

Since the final results will be obtained by entering an image captured by the device and transmitted to the laptop via USB cable in a processing algorithm, the device is dependent on the laptop, which facilitates the power supply of the development board, this being done via the first method listed above.

For the device created, we used two white LEDs with a diameter of 5mm and one supply voltage from 3.0V to 3.4V. They have the role of providing the same conditions of lighting for each photo taken. Thus the ambient light will not influence the image quality.

The button we used is of the “push” type without restraint, having the role of triggering the start of shooting. It is of the 90° THT type. Its dimensions are 7.1 × 7 mm and with a current of 50mA at 24V, but at a voltage less than 20 V the current will be 20 mA. The temperature range is from −30 °C to +65 °C.

**Figure 2:**
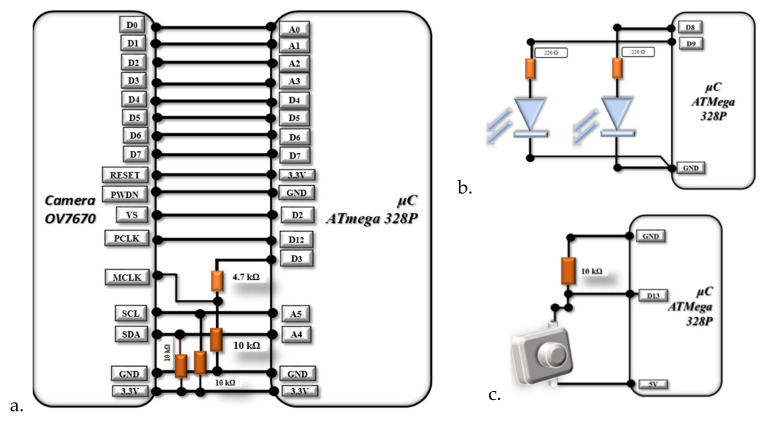
ATmega328P connection to: (**a**) OV7670 camera, (**b**) LEDs, (**c**) push button.

Figure 2a illustrates the connection of the OV7670 camera module to the Atmega328P microcontroller.

So the images are not to affected in terms of quality, we connected two LEDs on pins D8 and D9, as shown in Figure 2b. The two 220Ω resistors have the role of limiting the current through the LEDs [20].

Figure 2c shows the push-button connection with the role of triggering the start of shooting.

**Figure 3:**
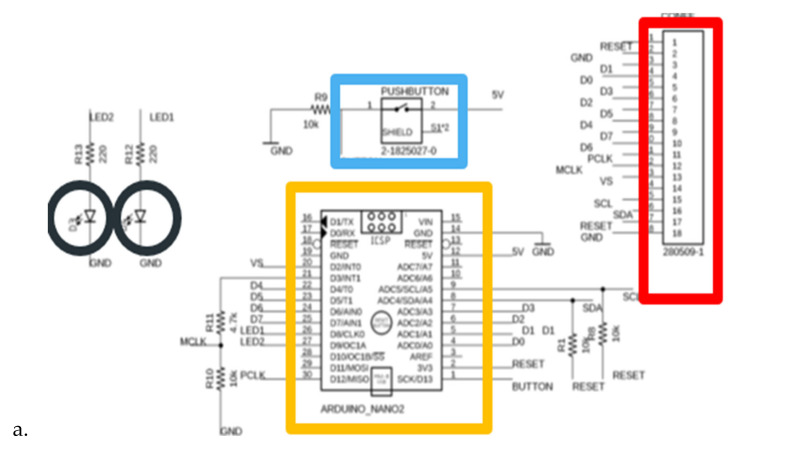

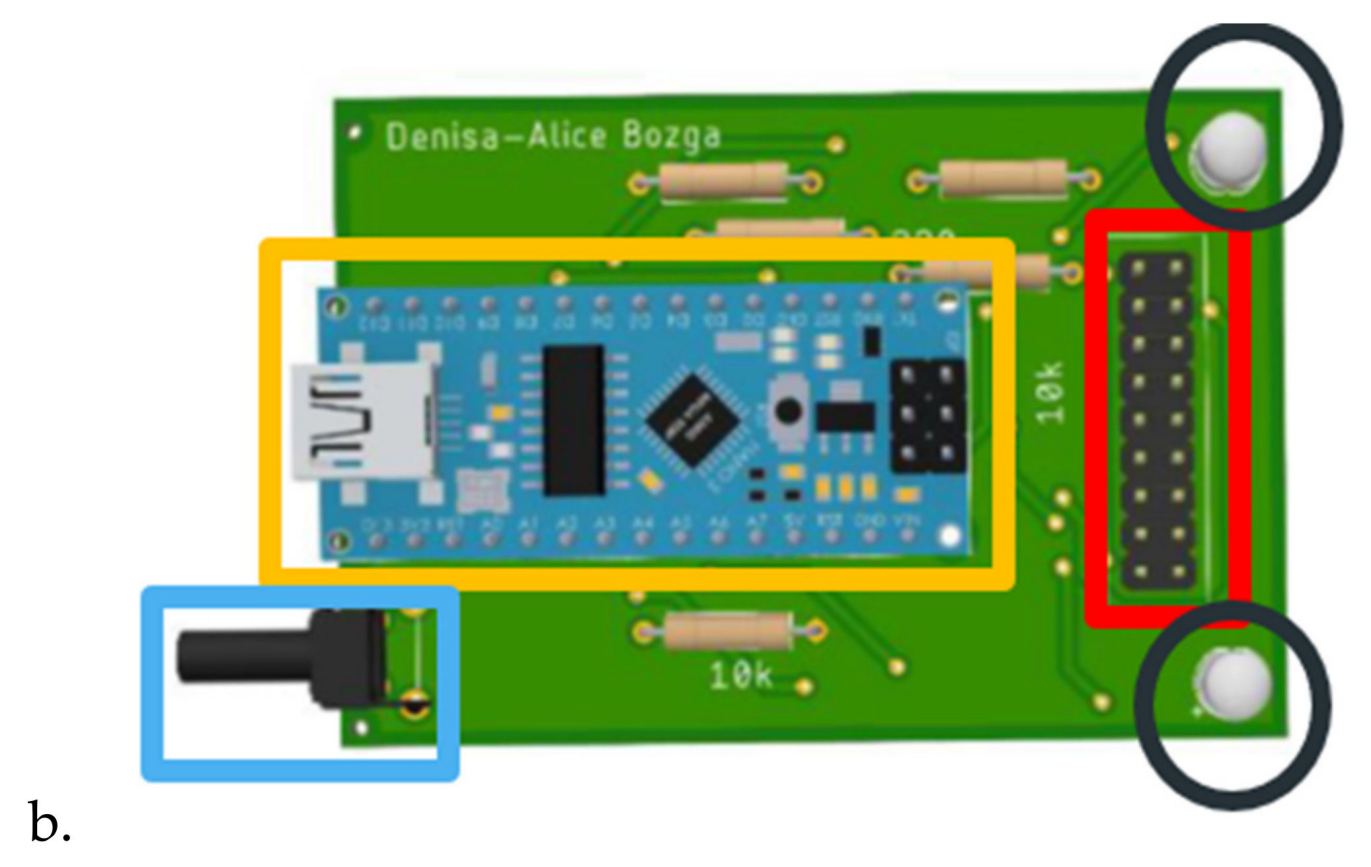
Electric design of the system in (**a**) in Eagle, (**b**) Fusion 360.

For the implementation of the printed circuit board, we used Eagle (Figure 3a). For visualization as close as possible to reality, we exported the 3D model in Fusion 360 (Figure 3b).

**Figure 4:**
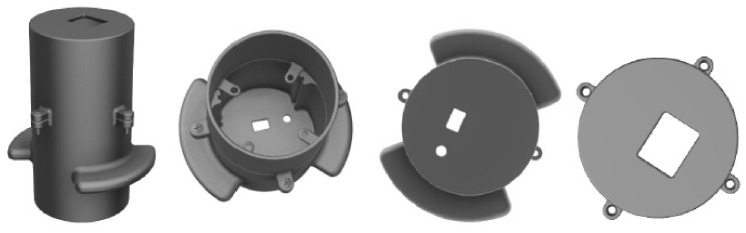
3D model of the device housing.

**Figure 5:**
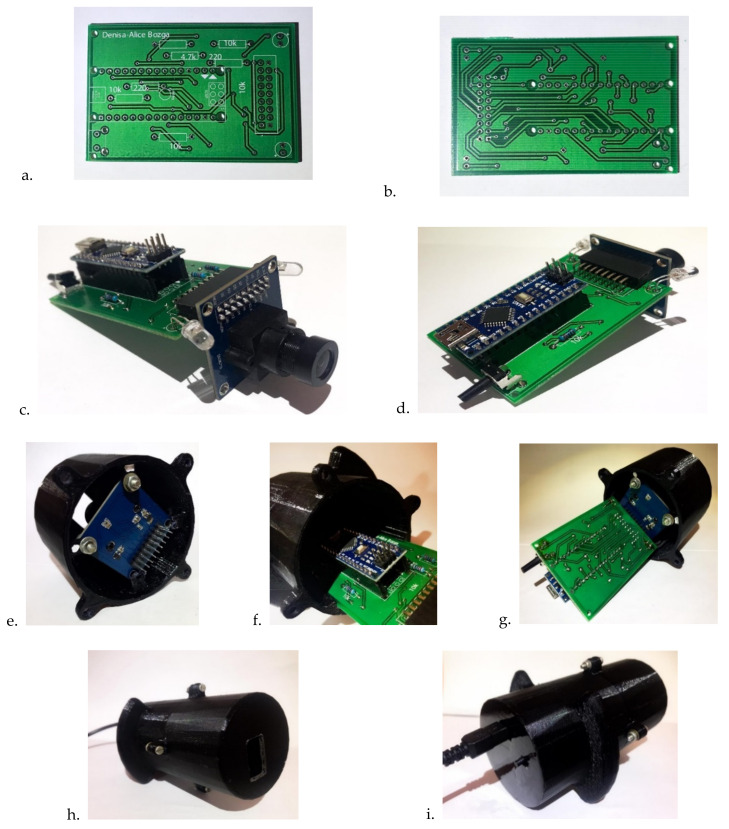
The final device: (**a**) PCB bottom side, (**b**) PCB top side, (**c**,**d**) populated PCB, (**e**–**g**) populated PCB mounted in the housing, (**h**) front-view of the mounted device, (**i**) back-view of the mounted device.

For the most straightforward use of the device and for obtaining images that are not influenced by external light, we created a 3D enclosure with the help of Fusion 360, a housing that allows connection of the previously presented components in a compact form. Figure 4 shows the 3D design of the enclosure for the final device. In Figure 5, the final device is presented.

The housing consists of two cylinders connected through the four screw types M3 × 14 mm. The height of the first cylinder (top) is 47.5 mm calculated according to the focusing distance of 25 mm, and the length of the camera lens of 20 mm is added to its 2.5 mm grip. The height of the second cylinder is 67.4 mm, this value being a sum of the populated PCB’s length and the length of the connectors that hold the OV7670 camera (Figure 5c,d). The bottom of the last cylinder has two cuts that facilitate access to the USB B of the Arduino Nano board for connection to the PC and the pressing of the push button that starts to capture the images. The top of the first cylinder has a lid with a cut of 20 × 15 mm because this is the image’s physical size that the camera can capture at a distance of 25 mm for focus. Thus, we ensured that wherever the mole is positioned within this cut will be captured by the camera.

### 2.3. Software Design

**Figure 6:**
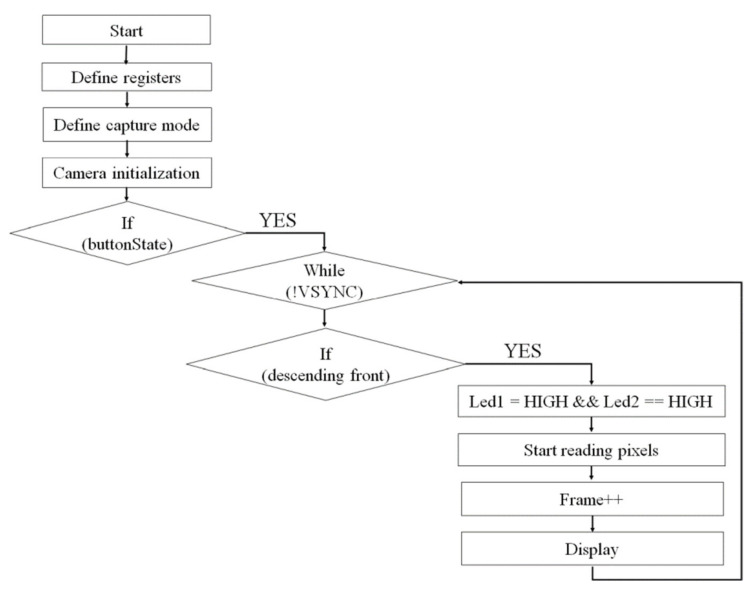
Image acquisition block diagram.

To exemplify the program implemented for image acquisition, we can follow its block diagram presented in Figure 6:

Saving images to the desired file from the laptop is done via a simple interface made in the IntelliJ programming environment in the Java language.

Through this interface, it is possible to acquire images by choosing the port to which the device is connected and choosing the Baud Rate, which is 115200. By multiple attempts with different values of the Baud Rate (for example 1000000, 57600, 38400) we noticed that its highest value for obtaining color images without imperfections is the one mentioned above.

**Figure 7:**
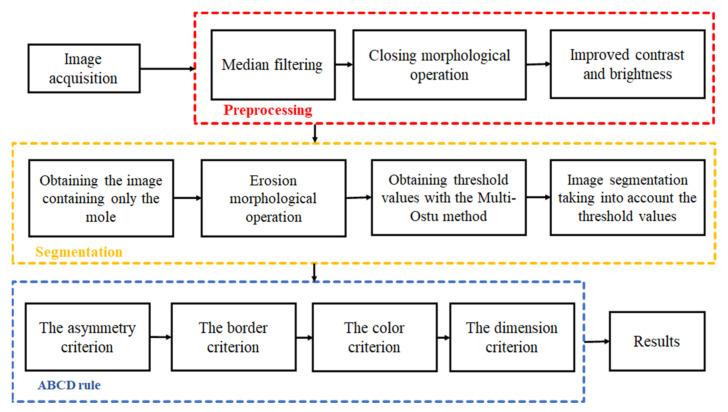
Block diagram for image processing.

To be able to follow the image review process more accessibly, see the following diagram (Figure 7).

The first step is image acquisition, and this is done by using the developed device. The images are uploaded for software processing, through an interface realized in Python programing language using the standard GUI (Tk GUI), OpenCv packages. Some improvements are applied to the images. The second step, preprocessing, involves correcting the image in terms of noise, brightness, and contrast to get the most eloquent results at the segmentation step. Changing the contrast is done with the help of point operations that are associations (mapping) that link the original gray level to its new value [21]. The enhancement of brightness and contrast was achieved with a grayscale clipping function (by a piecewise linear transformation). The median filter and the closing morphological operation were applied on the images to completely remove the noise and the hairs that may appear near the mole. Mathematical morphology takes a form-oriented approach to image processing. Appropriately used, mathematical morphology leads to processing that simplifies the structure of the image, preserving the essential characteristics of form and eliminating irrelevances.

The third step is image segmentation and its role is to detect the mole region. Segmentation is the division of the image into areas of interest according to specific criteria. The Otsu algorithm is commonly used to implement binarization, which is a method of classifying pixels into two classes according to the gray level. The Otsu thresholds are different for each image, depending on the grayscale intensities contained by the image. The limitations of this algorithm appear when it comes to a darker level of gray, and to eliminate errors that may occur we use the Multi-Otsu method. This method allows classification of pixels into multiple classes based on multiple threshold values using the same image.

In general, when an image containing a mole and segmentation is performed, it is divided into three classes. The first class is represented by the edges of the mole that represents the darkest part, the second class is the mole, and the last class is represented by the skin and is the lightest part. If the segmentation is carried out as described above, the algorithm needs two threshold values, and so we will obtain the background pixels, the intermediate pixels, and the lightest ones.

The ABCD rule [11] has played a major role in the dermatology field since 1985 when it was presented officially. It is based on several years of study and statistics on the four features (asymmetry, border, color, diameter) of moles. Based on this deep research the TDV (Total Dermatoscopic Value) parameter was established, where each ABCD criteria has a score and a factor of importance in deciding the diagnosis. The TDV value is used as a starting point in all skin evaluations by physicians and reveals whether further investigation is required.

To calculate the score, four ABCD criteria are considered:

○ Asymmetry-A—evaluates both shape and color distribution;
○ Border-B—evaluates contours that end abruptly, dividing the mole into eight segments;
○ Color-C—evaluates the presence of the six colors (white, red, light brown, dark brown, bluish-gray, black);
○ Diameter-D—evaluates that the mole diameter is larger than 6mm.

The description of the four criteria can be found in Table 1:

After finding the score offered by each criterion individually, the four values will be added and multiplied by the importance factor reported in Table 1. The ABCD-derived *TDV* (Total Dermatoscopic Value) score is obtained using (1):

(1) TDV=1.3∗A+0.1∗B+0.5∗C+0.5∗D

#### 2.3.1. Asymmetry

The parameter obtained by analyzing this criterion consists of two components. The first component refers to the percentage of non-overlap of the two parts of the image concerning the *X*-axis and concerning the *Y*-axis. The second component refers to the distribution of color on the moles’ surface.

After centering the image and rotating it, the non-overlap index Δ*A* is calculated according to (2):

(2) $$\begin{array}{c} NOVL(L,R)\leq T0\mathrm{and}\;NOVL(U,D)\leq T0\Rightarrow \Delta A=0\\ NOVL(L,R)\leq T0\mathrm{or}\;NOVL(U,D)\leq T0\Rightarrow \Delta A=1\\ \mathrm{Other}\Rightarrow \Delta A=2 \end{array}$$

where (*L*, *R*) means non-overlapping on the sides (left and right), (*U*, *D*) means non-overlap up and down, and *T*0 represents the threshold value for non-overlap and is considered as 5% of the mole area.

For the second component, the normalized histogram is made for each channel (R, B, and G) and for each part of the image (left–right, top–down).

After obtaining the two normalized histograms, the distance that aims to measure the differences between the two histograms called *Chi-Square* is calculated with (3):

(3) $$A_{color}=Chi_{square}=\sum_{i=1}^{n}\frac{{\left(hist_{1}(i)-hist_{2}(i)\right)}^{2}}{hist_{1}(i)+hist_{2}(i)}$$

Finally, the parameter for Asymmetry is obtained, which is calculated according to Equation (4):

(4) $$A=\frac{\Delta A+A_{color}}{2}$$

#### 2.3.2. Border

The border factor verifies the mole edges. To find out the irregularity of the edges of the mole, it is divided into 8 segments.

After defining the 8 segments, the standard deviation of each is calculated. If the calculated standard deviation exceeds the empirically chosen threshold value 15, then for the respective section the score will be 1.

For the final parameter indicating the score for the border criterion, all values obtained for each section are added.

#### 2.3.3. Color

To determine the color score, we check in the mole region the presence of the 6 colors mentioned in Table 2.

This is done using the Euclidean distance between each pixel in the mole region and color reference. In Table 2 are the values for the 6 reference colors. If the value of the distance calculated is less than or equal to the threshold value then the score that determines the color is increased by 1. The threshold value is calculated based on the distance between the highest reference and the lowest color reference. It should be noted that the color values presented in the table are normalized by 255.

#### 2.3.4. Diameter

**Figure 8:**
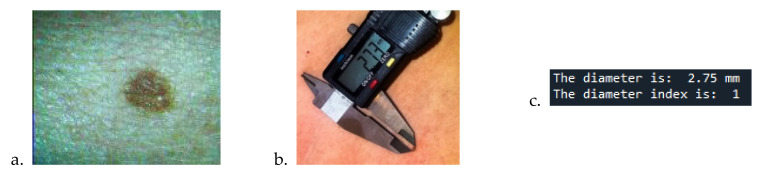
Mole diameter analysis for an image: (**a**) captured by the camera, (**b**) measured by vernier, (**c**) by calculating the diameter index.

Obtaining the parameter for the fourth criterion, diameter, is done using the X and Y axes of the mole region. It considers the resolution of the image in pixels and in real dots in mm (millimeters), due to the fact that our system is calibrated, as shown in Figure 8.

The image has a resolution of 160 × 120 pixels. The physical size at which the camera shoots is 25mm, this being the distance of focus to the mole, and the physical size of the image is 20 × 15 mm.

Checking which of the two variables, the *X* or *Y* axes of the mole region, is larger, the correlation between the number of pixels in the mole region, resolution, and physical size is made according to (5) to compute the mole dimension in mm. The diameter score is obtained considering the *M* value:

(5) $$nr_{X}>nr_{Y}\Rightarrow M=\frac{nr_{X}*15mm}{120}\;nr_{X}<nr_{Y}\Rightarrow M=\frac{nr_{Y}*20mm}{160}$$

## 3. Experimental Results

To validate our system for segmentation and computing of ABC parameters, we used the PH2 dataset [22], which contains 200 moles, including 40 melanomas, 80 common moles, and 80 atypical moles. The validation was carried out just for the segmentation step, and some of ABCD rule scores are freely available online. The diameter score was not validated because we did not correlate the pixels and mm values for the PH2 dataset images.

Next, we used our system to capture images and analyze them.

To facilitate the use of the device created and the reading of the results by any user, the final software package contains two executable files that allow interaction between the PC, the device, and the end-user without the need for anything additional.

**Figure 9:**
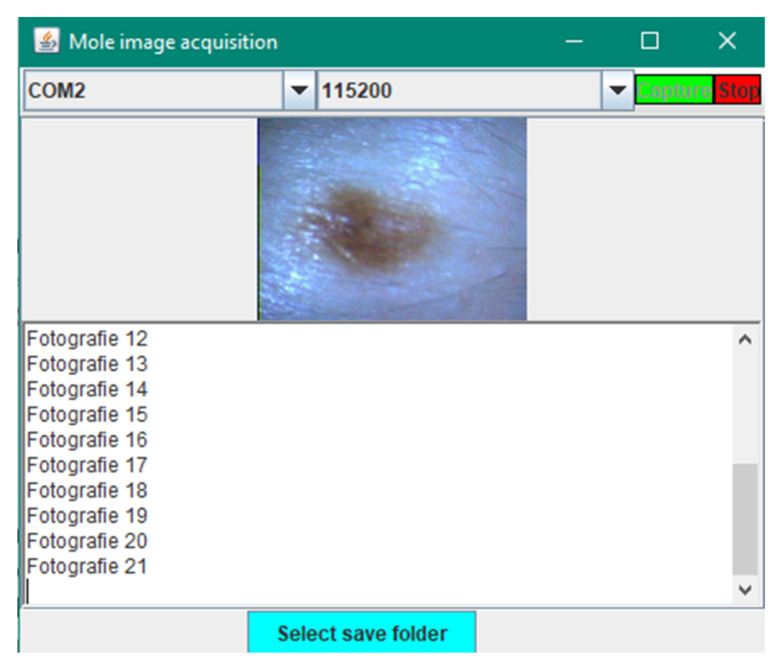
Interface for image acquisition.

Once the first executable is launched, a window will open that will permit choice of the port on which the connection is made, selection of the Baud Rate and the start of capturing the image, as shown in Figure 9.

Using the developed device, we captured mole images for patients who also took a medical examination by a specialist. The instrumentation used by the doctor was the standard dermatoscope, which is a quick, non-invasive and painless method of examination that allows the examination of both the skin surface and the slightly deeper structures and is used in combination with a medical history and clinical eye examination. It is recommended to be performed by doctors with adequate training in this technique. Thus, our database contains 26 images for which we have a diagnosis given by a specialist.

**Figure 10:**
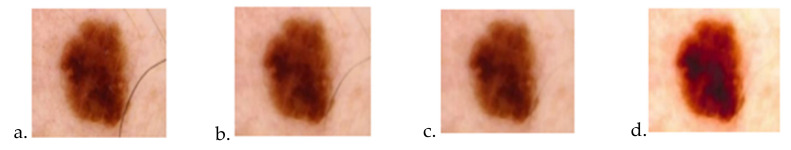
The results after the first stage of image processing: (**a**) image for analysis, (**b**) image after the median filter, (**c**) image after morphological operations, (**d**) image after improvement of brightness and contrast.

The results obtained after the first stage of image processing are presented in Figure 10.

**Figure 11:**
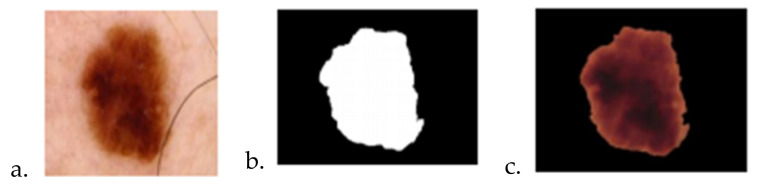
The result after Multi-Otsu: (**a**) image for analyze, (**b**) Image after segmentation, (**c**) Image after Multi-Otsu.

**Figure 12:**
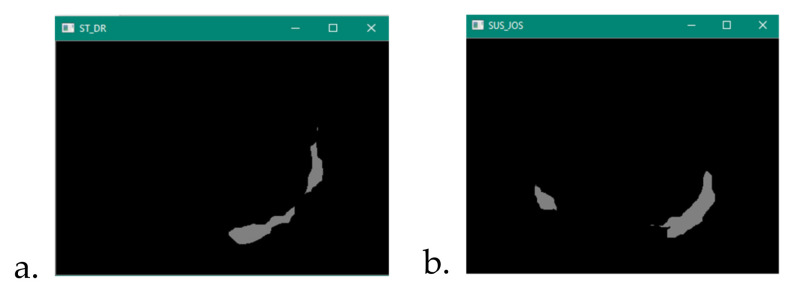
Non-overlap results of Asymmetry between (**a**) left and right, (**b**) top and bottom.

The results after the segmentation performed with the Multi-Otsu method are presented in Figure 11:

The results obtained following the asymmetry criterion is presented in Figure 12:

The values computed on the currently processed image (Figure 10a) by applying the ABCD rule and the final score for each criterion are presented in Table 3.

After the four criteria scores from the ABCD rule, the value of the TDV can be computed. Depending on the value of the TDV, a final result is established according to Table 4.

The interface allows selection of the image to be analyzed and its display together with the final results.

**Figure 13:**
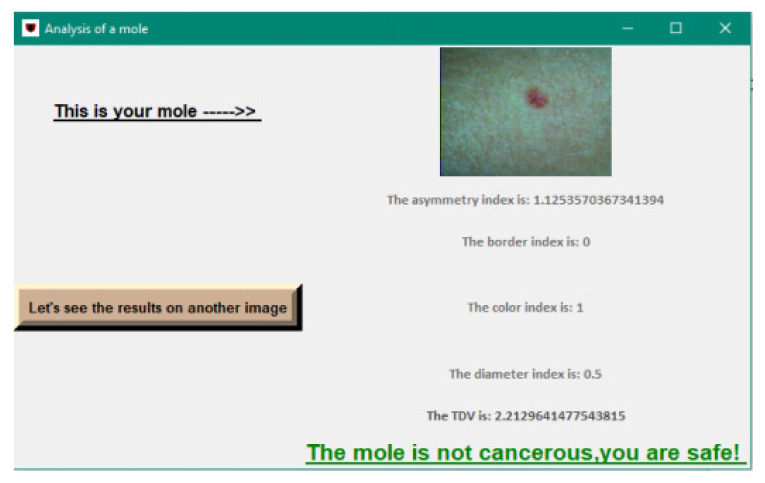
Final interface.

Figure 13 shows the interface after choosing an image to analyze.

In Table 5 are presented the ABCD rule scores and the diagnosis. We considered for exemplification four images, two with non-cancerous moles, one with cancerous mole, and one with a possibly cancerous mole that a doctor should evaluate.

The results in our database, with 26 images captured via our developed device, are summarized in Table 6 and Table 7. The collaboration with the doctor helped us to compare the diagnosis results. The doctor, with the use of a dermatoscope, classified 24 moles as non-cancerous, one as cancerous, and one as possibly cancerous. From the 24 moles classified by the doctor as non-cancerous, our system considered two as possibly cancerous. By comparing the results with the two evaluation methods (doctor vs. our device), we can underline that our system will classify the border cases as possibly cancerous, which can be considered a positive by suggesting to the end-user a possible problem. Therefore, at the evaluation of our system we have the values for TP (True Positive), TN (True Negative), FP (False Positive) and FN (False Negative) for not-cancerous moles classification task, as: TP = 22.0; TN = 2.0; FN = 2.0, FP = 0.0. The overall performance measures obtained using our device are—Accuracy: 0.92, Precision: 1.0, Re-call: 0.92, F1-score: 0.96. We can see that the Precision score is 1, which means that the non-cancerous moles are all classified correctly with no false-positive classifications, and Recall being less than one (0.92) means that the system prefers to have false-negative detection (detected as possible cancerous moles even if that are not) so that the patient can verify these moles with doctor assistance.

The confusion matrix is as follows, where the classes are C1 = Not Cancerous, C2 = Cancerous, C3 = Possibly Cancerous (the values are illustrated also in Table 6.):

## 4. Conclusions

We can underline that skin cancer is the most commonly known disease of the largest human body organ, the skin. It is also one of the most common types of cancer, with an alarming increase globally.

Multiple studies show that this disease can either be treated or prevented if it is discovered at an early stage or if a regular medical examination is carried out.

The role of the created device is to facilitate the analysis of moles, even if this is performed for guidance and not from a medical point of view. The final diagnosis is undoubtedly the responsibility of a specialized doctor. However, the ABCD rule based on which the processing was performed is most often used in such analyzes and offers an accuracy of 92.8%.

The primary purpose was to create the moles’ images without being affected by any external factor because this could influence the analysis. Thus, we designed the enclosure of the device in the shape of a cylinder that prevents the entrance of ambient light. The choice of the OV7670 camera with CMOS image sensor was also made to take photos as accurately as possible, the module allowing this due to its multiple functions. The device’s dimensions were an aspect that we considered when creating it, out of the desire to make it small and easy to handle by the user via the two interfaces.

The diagnosis proposed by our device is close to what a doctor can give, demonstrated with the comparative analysis taken on our database. Our device will classify the border cases, between a healthy mole and a suspicious one, as probably problematic. In this case, the end-user should seek a specialist investigation at an early stage of the mole.

Thus, we obtained a final device that, as we intended, provides the results of image analysis of a mole on the skin through a friendly and easy-to-use interface.

Further testing will be made in specialized dermatology hospitals to improve the device.

## Figures and Tables

**Table 1:** Criteria for the ABCD rule [11].

Criteria	Score	Factor of Importance
A	0–2	x1.3
B	0–8	x0.1
C	1–6	x0.5
D	0.5–5	x0.5

**Table 2:** References of the six colors [12].

White	(1, 1, 1)	(0.9608, 0.9608, 0.9608)	(0.9216, 0.9216, 0.9216)	(0.8824, 0.8824, 0.8824)	(0.8431, 0.8431, 0.8431)	(0.8039, 0.8039, 0.8039)
Black	(0, 0, 0)	(0.0392, 0.0392, 0.0392)	(0.0784, 0.0784, 0.0784)	(0.1176, 0.1176, 0.1176)	(0.1569, 0.1569, 0.1569)	(0.1961, 0.1961, 0.1961)
Red	(0.7843, 0.5882, 0.3922)	(1,0.1961, 0.1961)	(0.7843, 0, 0)	(0.7843, 0.1961, 0.1961)	(0.5882, 0, 0)	(0.5882, 0.1961, 0.1961)
Light brown	(0.7843, 0.5882, 0.3922)	(0.7843, 0.3922,0)	(0.7843, 0.3922, 0.1961)	(0.5882, 0.3922, 0.1961)	(0.5882, 0.3922, 0)	(0.5882, 0.1961, 0)
Dark brown	(0.5882, 0.3922, 0.3922)	(0.4902, 0.2941, 0.2941)	(0.3922,0.1961, 0.1961)	(0.3922, 0.1961, 0)	(0.3922, 0,0)	(0.1961, 0,0)
Bluish gray	(0.5882, 0.4902, 0.5882)	(0.4902, 0.4902, 0.5882)	(0.3922, 0.3922, 0.4902)	(0.3922, 0.4902, 0.5882)	(0.1961, 0.3922, 0.5882)	(0, 0.3922, 0.5882)

**Table 3:** Results for: Asymmetry, Border, Euclidian distance of each color, Final score.

**Results of the Asymmetry:**								
Non-overlapping between left and right				Non-overlapping between top and bottom		Chi_square	Asymmetry score	
1925				2340		0.0262594	1.013129	
**Results from border:**								
S1	S2	S3	S4	S5	S6	S7	S8	Border score
5.8472	6.5147	6.06682	6.4209	3.31381	1.5999	7.8649	13.8412	0
**Calculation of euclidean distance for each color:**								
CX1		CX2		CX3	CX4		CX5	CX6
1.165395		0.438254		0.376436	0.344173		0.285576	0.626262
**Calculation of threshold values and final score of the criterion:**								
T0_WHITE	T0_BLACK	T0_RED		T0_LIGHT_BROWN	T0_DARK_BROWN		T0_ GRAY	Color score
0.339655	0.339655	0.818156		0.588233	0.679252		0.415973	3

**Table 4:** Assumption and Results.

Assumptions	Results
TDV < 4.75	The mole is not cancerous.
4.75 ≥ TDV ≤ 5.45	The mole presents a cancer risk.
TDV > 5.45	The mole is cancerous.

**Table 5:** ABCD rule scores with the diagnosis for four images.

Images captured with the device	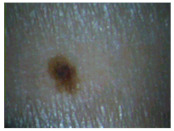	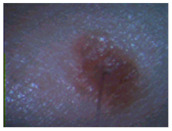	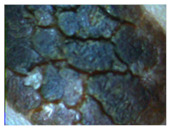	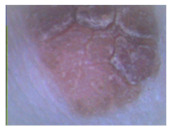
Asymmetry	1.19	1.32	1.61	1.38
Border	0	0	4	1
Color	2	3	3	3
Dimension	1	3	5	4
TDV	3.05	4.71	6.49	5.39
Diagnostic	“The mole is not cancerous, you are safe!”	“The mole is not cancerous, you are safe!”	“The mole is cancerous and must be checked by a doctor!”	“The mole could be cancerous, it should be checked by a doctor!”

**Table 6:** Confusion matrix.

	C1	C2	C3
C1	22	0	2
C2	0	1	0
C3	0	0	1

**Table 7:** Classification results on our database (26 captured images).

Classification Method	The Number of Images Classified as:		
	Not Cancerous	Cancerous	Possibly Cancerous
Doctor with dermatoscop	24	1	1
Our Device	22	1	3

## Data Availability

Not applicable.
